# Ischemic Stroke due to Intracranial Embolization of a Pelvic Phlebolith in a Pregnant Patient Successfully Treated by Surgical Embolectomy following Attempted Endovascular Thrombectomy

**DOI:** 10.1155/2023/1653631

**Published:** 2023-12-06

**Authors:** Sherwin Azad, John Anson, Tamara Majic, Raisa Lev, Varoujan Kostanian

**Affiliations:** ^1^HCA Mountainview Hospital Sunrise Health GME Consortium, Department of Radiology, USA; ^2^Sunrise Hospital and Medical Center, USA

## Abstract

An unusual case of ischemic stroke due to calcified cerebral embolus occurring in a pregnant patient during the peripartum period is reported. The source of the embolus was suspected to be a pelvic phlebolith in origin which paradoxically embolized via a patent foramen ovale to the supraclinoid right internal carotid artery. To our knowledge, this is the first reported case of calcified cerebral embolus attributed to paradoxical embolism of a pelvic phlebolith, and we theorize that introduction of the phlebolith into the venous system may have occurred as a consequence of vascular remodeling due to pregnancy-related hemodynamic changes. Clinicians should be aware of this potential source of calcified cerebral emboli in patients with a patent foramen ovale during pregnancy. Our patient ultimately achieved an excellent outcome with surgical endarterectomy and embolectomy following an unsuccessful attempt at mechanical thrombectomy.

## 1. Introduction

Calcified cerebral embolus is a rarely reported cause of cerebral ischemic infarction, with mixed data on the effectiveness of current interventional treatments such as mechanical thrombectomy [1–3]. Here, we report an unusual case of calcified cerebral embolus occurring in a pregnant female during the peripartum period. We suspect that this occurred due to paradoxical embolization of a pelvic phlebolith via a patent foramen ovale, a mechanism of stroke that has not been reported previously.

## 2. Case

**Figure 1:**
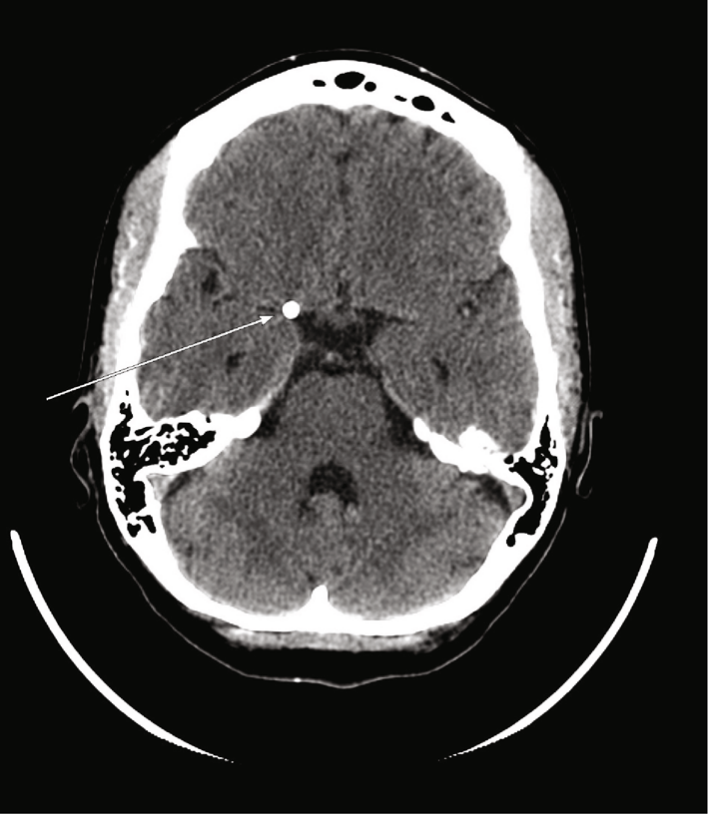
Noncontrast axial CT head demonstrates a rounded calcification (arrow) within the right carotid terminus.

**Figure 2:**
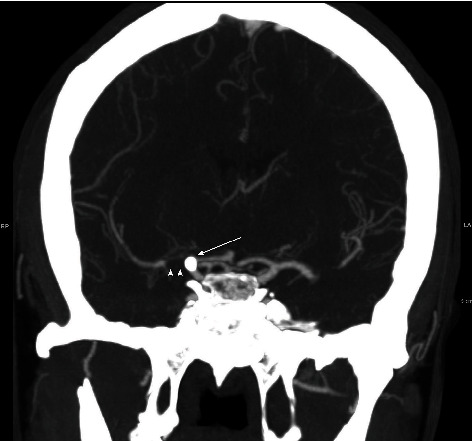
CT angiography of the head. Coronal maximum intensity projection (MIP) demonstrates occlusion of the right carotid terminus (arrow) due to calcified cerebral embolus and absent flow within the right M1 middle cerebral artery (arrowheads).

**Figure 3:**
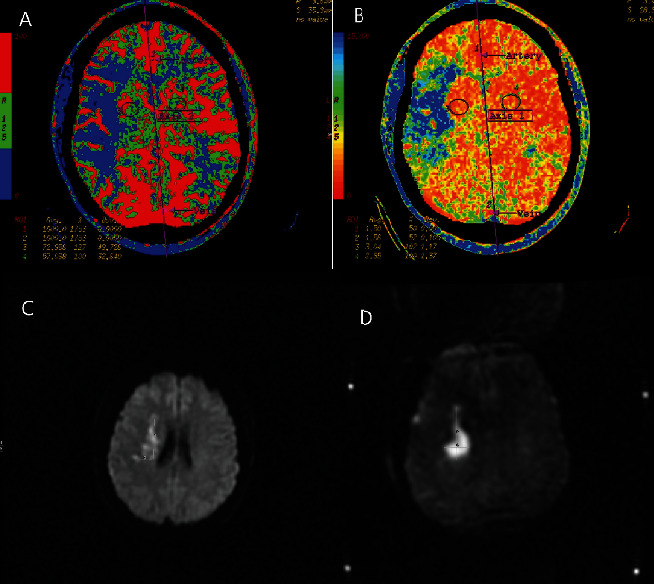
(a–d) CTA perfusion was obtained approximately 14 hours following presentation, following delivery by cesarean section. Perfusion maps demonstrate reduced blood flow (a) and reduced mean transit time (b) in the right MCA territory. Initial MRI (c) obtained within 3 hours following presentation demonstrates a focus of diffusion restriction in the right basal ganglia and corona radiata, measuring up to 4 cm compatible with core ischemic infarct. Postprocedure MRI (d), obtained after attempted thrombectomy and surgical endarterectomy, approximately 48 hours from the prior MRI, demonstrates stable 4 cm core infarct with sparing of the majority of the at-risk territory.

**Figure 4:**
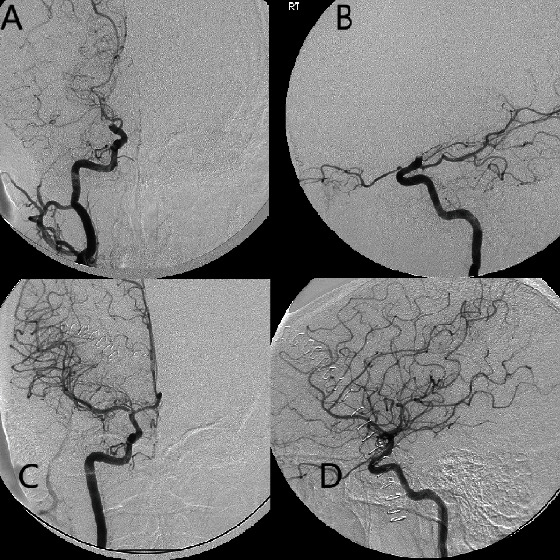
(a–d) Digital subtraction angiography, arterial phase, and frontal and lateral views, obtained during injection of the right internal carotid artery (a, b) pre- and post-endarterectomy and embolectomy (c, d), demonstrate complete occlusion of the right internal carotid artery at the terminus (a, b), with complete recanalization (TICI 3) following endarterectomy and embolectomy (c, d). There is metallic subtraction artifact due to aneurysm clip overlying the carotid terminus on frontal view (c).

**Figure 5:**
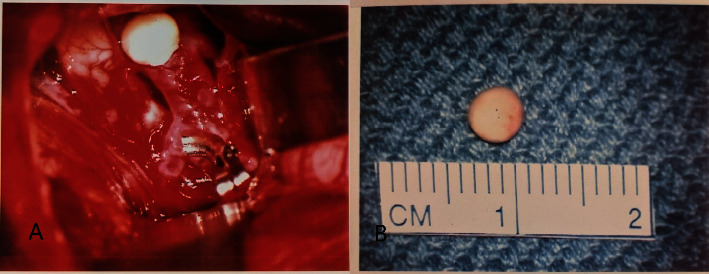
(a) Intraoperative view of the calcified embolus in the carotid terminus. (b) Gross specimen of the calcified embolus removed at surgery.

**Figure 6:**
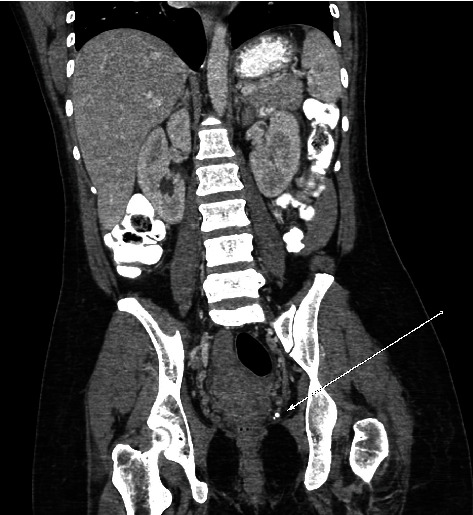
Contrast-enhanced CT of the abdomen and pelvis, coronal view, demonstrates calcified phleboliths in the left pelvis (arrow) measuring up to 5 mm, similar in size and morphology to the calcified cerebral embolus.

The patient was an otherwise healthy, pregnant female in her early 30s at 37 weeks gestation who presented in 2009 with symptoms of acute stroke including left-sided facial droop, dysarthria, with left-sided weakness and numbness. Noncontrast CT of the head was obtained demonstrating a rounded calcification at the right carotid terminus (Figure 1). MRI/MRA brain, obtained approximately 3 hours after presentation, demonstrated a 4 cm focus of diffusion restriction within the right basal ganglia and corona radiata within the right middle cerebral artery (MCA) territory and loss of flow void at the distal right M1 MCA. Labor was induced, and delivery of the patient's fetus was performed via cesarean section. Following the uncomplicated delivery, CT angiography and CT perfusion were performed demonstrating a 5 mm rounded, calcific density within the supraclinoid right internal carotid artery (ICA) (Figure 2) and occlusion of the proximal right M1 MCA with narrowing at the MCA trifurcation and a large right MCA perfusion deficit and penumbra (Figure 3). Catheter-based cerebral angiography was performed approximately 30 hours following the initial presentation, demonstrating complete occlusion of the right internal carotid artery terminus and right M1 MCA (Figures 4(a) and 4(b)). After successfully crossing the lesion, mechanical thrombectomy was attempted utilizing a combination of balloon sweeps and simultaneous aspiration via the balloon-guiding catheter; however, this was unsuccessful after a total of 5 attempts. Given the large territory at risk in this young, otherwise healthy patient, the patient was emergently taken to the operating room straight from the angio suite, and surgical endarterectomy and embolectomy were performed via a right-sided craniotomy. A rounded calcified embolus was removed (Figure 5), and there were no immediate surgical complications, with postoperative angiography demonstrating complete recanalization TICI 3 (Figures 4(c) and 4(d)). Additional investigations revealed a patent foramen ovale and calcified phleboliths in the pelvis (Figure 6); no calcified arterial plaque or other potential source of calcified emboli was identified. Her follow-up MRI the day following surgical embolectomy, approximately 2 days after her initial presentation, showed approximately stable size of the core infarct with sparing of the majority of at-risk right MCA territory (Figure 3). She was maintained on dual antiplatelet therapy for 6 months postprocedure, and following recovery from her stroke, the patent foramen ovale was closed. Her neurological outcome was excellent, with resolution of almost all deficits, although she reported being easily fatigued when returning to her normal job duties at the four-month follow-up visit. Her neurological outcome was consistent with a modified Rankin scale of 1 for fatigue.

## 3. Discussion

To our knowledge, this is the first reported case of cerebral ischemic infarction suspected to have occurred due to paradoxical intracranial embolism of a calcified pelvic phlebolith. In our pregnant patient, we theorize that pregnancy-related changes in vascular flow and hemodynamics within the pelvis may have resulted in the introduction of the phlebolith into the venous system, with paradoxical embolus then occurring via a patent foramen ovale. During pregnancy, venous remodeling and expansion of collateral circulation may be induced by a number of hemodynamic changes including vasodilation due to hemodilution, increased blood supply and venous drainage required for supply to the fetus, as well as venous compression by the gravid uterus [4].

Calcified cerebral emboli are a rare cause of ischemic stroke that have historically been infrequently reported, with only 48 cases reported in total prior to 2014, and remain a diagnostic challenge as they are frequently misinterpreted on diagnostic imaging [1]. In recent retrospective reviews of large, multicenter, mechanical thrombectomy registries, calcified cerebral emboli were found to account for 1.3% and 1.8% of strokes in patients who underwent mechanical thrombectomy, [2, 3] leading to a suggestion that calcified cerebral emboli may be underreported and underrecognized as a cause of ischemic stroke. Notably, the source of the embolus is undetermined in many cases, with one of the largest reviews to date reporting that the source is undetermined in 37.5% of the patients identified [2].

Maurer et al. found that patients with stroke due to calcified cerebral emboli had worse angiographic and clinical outcomes following mechanical thrombectomy as compared with patients with noncalcified emboli [2]. The MR CLEAN investigators reported equivalent functional outcomes, despite worse angiographic results and worse improvement in NIHSS score, and a statistically significant association between the use of stent-retriever devices and successful recanalization [3]. This is an interesting development, as earlier reports demonstrated poor success rates with the use of stent-retrievers, as well as poor mortality rates and functional outcomes following attempted treatment [5]. Treatment with TPA has been reported as less effective in patients with CCE [2, 3, 6]. Surgical embolectomy of calcified cerebral embolus has rarely been reported; however, excellent outcomes are possible [7]. A recent article by Fiedler et al. demonstrated that microsurgical intervention, including embolectomy, bypass, or a combination of the techniques, may be a promising third-tier treatment in patients with persistent large vessel occlusion and failed endovascular thrombectomy, not limited to only calcified emboli [8].

A significant limitation of our reported case is that patient selection and attempts at endovascular thrombectomy were limited by the available devices and imaging techniques at the time, which are not comparable to modern devices and techniques. Additionally, our decision algorithm was further complicated by a lack of evidence-based recommendations at the time, and the decision to induce labor by C-section, which resulted in a significant time interval from her initial presentation to eventual reperfusion. Despite this, our patient ultimately achieved an excellent outcome.

## 4. Conclusion

Stroke due to a calcified cerebral embolus is an infrequently encountered clinical scenario that requires a high index of suspicion for prompt and accurate diagnosis, involves unique treatment considerations, and is frequently associated with an undetermined source of embolus. To our knowledge, this is the first reported case of calcified cerebral embolus thought to have occurred due to paradoxical embolism of a pelvic phlebolith via a patent foramen ovale. Clinicians should be aware of this potential source of calcified cerebral emboli in patients, especially during pregnancy.
